# The Efficacy of MSC-Derived Exosome-Based Therapies in Treating Scars, Aging and Hyperpigmentation: A Systematic Review of Human Clinical Outcomes

**DOI:** 10.3390/reports8040268

**Published:** 2025-12-17

**Authors:** Fawwaz F. Al Shammrie, Lama Z. Alhemshy, Maitha M. Althawy, Maryam M. Alfaraj, Aseel S. Alotaibi, Danah S. Alali, Omar H. Alsaggaf, Layan Z. Alhamashi, Lama M. Albelowi

**Affiliations:** 1 Department of Dermatology, College of Medicine, University of Hail, Hail 55472, Saudi Arabia; fawwzf@yahoo.com; 2 College of Medicine, University of Hail, Hail 55424, Saudi Arabia; 3 College of Medicine, Batterjee Medical College, Jeddah 21442, Saudi Arabia; 4 College of Medicine, Imam Abdulrahman Bin Faisal University, Dammam 32662, Saudi Arabia; 5 College of Medicine, Majmaah University, Majmaah 11952, Saudi Arabia; 6 College of Medicine, King Faisal University, Mubaraaz 36341, Saudi Arabia; 7 College of Medicine, King Abdulaziz University, Jeddah 23452, Saudi Arabia; 8 College of Medicine, Taibah University, Al-Madinah Al-Munawwarah 42381, Saudi Arabia

**Keywords:** mesenchymal stem cell, exosome, scar, hyperpigmentation, skin aging

## Abstract

**Background:** Recent advancements in regenerative medicine have introduced mesenchymal stem cell–derived exosomes (MSC-Exos) as a novel therapeutic approach. Exosomes are extracellular vesicles containing proteins, lipids, and RNAs capable of modulating cellular behavior and promoting tissue regeneration. A systematic review of human studies is warranted to summarize outcomes, assess therapeutic value, and guide clinical applications. **Objectives:** This systematic review synthesizes current evidence on mesenchymal stem cell–derived exosomes for cutaneous scars, aging, and hyperpigmentation, with a focus on functional and aesthetic outcomes. **Method:** A comprehensive search of PubMed, Scopus, Embase, Web of Science, and Google Scholar (January 2010–July 2025) was performed following 2020 PRISMA guidelines. Eligible studies included studies that were randomized controlled trials, pilot studies, case series, and case reports involving human participants treated with MSC-Exos. Outcomes assessed were scar remodeling, pigmentation, skin regeneration, recurrence, and adverse events. Data extraction and bias assessment were conducted independently. **Result:** Six studies (*n* = 99; age 19–72 years) from diverse regions, including the United States, the Republic of Korea, and México, were included. MSC-Exos therapy showed promising improvements in reducing scar thickness (32.5% vs. 19.9%, *p* < 0.01), wrinkle parameters were reduced by 1 (2.4–14.4% vs. 6.6–7.1%, *p* < 0.05), and elasticity was enhanced (+11.3% vs. −3.3%, *p* = 0.002) Additional benefits included hydration (+6.5% vs. +4.5%, *p* = 0.37) and reduced melanin index (−9.9% vs. −1%, *p* = 0.44). The Global Aesthetic Improvement Scale score showed significant improvement (*p* = 0.005). Using the Investigator Global Assessment, 16 out of 25 areas treated with exosomes showed significant improvement (grade ≥ 2), compared to 12 out of 25 areas in the control group (*p* = 0.02), indicating that exosome treatment led to more visible improvement. Complete resolution of icepick scars, partial improvement of boxcar/rolling scars, and no recurrence of keloids (18/21) were reported. Adverse events were mild and transient. **Conclusions:** Early human evidence suggests that MSC-Exos may offer potential therapeutic benefits for scars, hyperpigmentation, and skin aging, with favorable short-term safety profiles. However, the current evidence remains preliminary due to small sample sizes, heterogeneous study designs, and limited follow-up durations. Larger, well-designed randomized trials are needed to confirm long-term efficacy and safety.

## 1. Introduction

Skin is composed of several distinct layers and a variety of cell types, each of which has a crucial role in its function as the external shield of the body. The skin is composed of the epidermis, dermis, and hypodermis, arranged in a well-organized structure. Different cell types are found within these layers, including epithelial cells, keratinocytes, and melanocytes, as well as networks of blood vessels, lymphatic vessels, and nerve fibers [1].

During scar formation, the epidermis and dermis of the affected skin part are replaced by an abnormal connective tissue due to an impaired wound-healing process [2]. This healing process is regulated through a complex network of organized cell-to-cell interaction, growth factors, cytokines, and extracellular matrix. There are two general categories of scars: normal and pathological. Normal scar tissue initially consists of loosely organized fibrous connective tissue, which gradually undergoes remodeling to form a stronger and more structured matrix. In contrast, pathological scars, which include hypertrophic, keloid, and atrophic scars, result from impaired healing processes [3]. Hyperpigmentation is skin darkening that occurs when melanocyte activity or distribution is disturbed. It can be linked to exposure to UV light, hormonal fluctuations, inflammation, and certain medications [4]. Skin aging is characterized by fine wrinkles, loss of elasticity, and skin dryness and is attributed to reduced collagen synthesis and subsequently reduced collagen levels [5]. Collectively, these dermatological changes may lead to emotional concerns and aesthetic dissatisfaction [4,5].

Exosomes are nanosized extracellular vesicles that are released from cells to facilitate intracellular communication and transport proteins, lipids, mRNA, and microRNAs [6,7]. Exosomal contents have the capacity to modify a variety of cellular processes once they are taken up by the target cells. The functional impact of exosomes completely depends on their origin [6]. Exosomes originating from damaged or stressed cells carry pro-inflammatory cytokines, microRNAs, and oxidative stress signals that can promote endothelial dysfunction or fibrosis [8].

On the other hand, exosomes derived from a healthy source, particularly mesenchymal stem cell (MSC)-derived exosomes, have shown promising efficacy in treating various dermatological disorders like hyperpigmentation, aging, and scarring [9]. It is because they provide regenerative and immunomodulatory qualities of MSCs, but with no risk of tumor formation or immune rejection, as in MSCs alone [7]. Exosomes facilitate wound healing and re-epithelialization and boost the dermal matrix reconstruction by gene expression regulation and protein production [7]

Due to the scarcity of medical research about the efficacy of MSC-derived exosomes in dermatological disorders, this systematic review aims to address an important gap in knowledge. By analyzing the potential behind MSC-derived exosomes in the treatment of scars, hyperpigmentation, and skin aging, this study offers the most evidence to date and sets the stage for future clinical applications of MSC-derived exosome therapy in dermatology.

## 2. Methods

### 2.1. Protocol Registration

This systematic review was written according to the PRISMA 2020 guidelines [Table S1] and was registered with PROSPERO under the registration number CRD420251108123 [10]. Ethical approval was not applicable, as this review synthesized published data without direct involvement of human subjects.

### 2.2. Search Strategy

A comprehensive search of the electronic databases PubMed, Scopus, Embase, Web of Science, and Google Scholar was conducted to identify all relevant studies published from January 2010 to July 2025. For Google Scholar, only the first 200 results were screened. The search strategy incorporated a combination of keywords and MeSH terms related to “Mesenchymal Stem Cell,” “MSC,” “MSCs,” “Exosome,” “Extracellular Vesicle,” “MSC-Exos,” “MSC-EVs,” “Scar,” “Cutaneous Scar,” “Atrophic Scar,” “Hypertrophic Scar,” “Keloid,” “Hyperpigmentation,” “Skin Aging,” and “Wrinkles.” Boolean logic operators were applied to ensure the retrieval of studies evaluating MSC-derived exosomes or extracellular vesicles in the management of scars, aging, and wrinkles (“Mesenchymal Stem Cell” OR “MSC” OR “MSC-derived” OR “Mesenchymal Stromal Cell”) AND (“Exosome” OR “Extracellular Vesicle” OR “EV” OR “MSC-exosomes” OR “MSC-EV”)AND (“Scar” OR “Atrophic Scar” OR “Acne Scar” OR “Hypertrophic Scar” OR “Keloid” OR “Skin Aging” OR “Wrinkles” OR “Hyperpigmentation”) AND (“Clinical Trial” OR “Human Study” OR “Randomized Controlled Trial” OR “RCT” OR “Pilot Study” OR “Case Series” OR “Case Report”). This search was guided by a PICO framework in which (P) included human participants with cutaneous conditions such as scars, aging, or hyperpigmentation; (I) included topical or injectable MSC-derived exosome therapies delivered through various clinical techniques; (C) included placebo, saline, no treatment, split-face controls, or standard dermatologic therapies; and (O) included improvements in scar parameters, pigmentation, wrinkles, elasticity, hydration, healing response, and recurrence rates. In addition, the reference lists of all included studies were manually screened to identify any additional eligible articles not captured through the database searches.

### 2.3. Inclusion and Exclusion Criteria and Screening

Three authors independently conducted the title and abstract screening using criteria included (1) studies reporting on human participants of any age and gender treated with mesenchymal stem cell (MSC)-derived exosome therapies for cutaneous scars, aging, or hyperpigmentation; (2) randomized controlled trials (RCTs), split-face RCTs, prospective studies, pilot studies, case series, and case reports (3) studies reporting on outcomes relevant to the review objectives (e.g., scar thickness, pigmentation, wrinkles, elasticity, hydration, texture, patient satisfaction); and (4) studies published in English. Exclusion criteria included (1) non-English language studies; (2) editorials, letters, commentaries, reviews, or conference abstracts; (3) in vitro studies or animal studies; (4) studies not using MSC-derived exosome therapy as an intervention; (5) studies not reporting outcomes relevant to the study objectives. Full-text articles of potentially eligible studies were then retrieved and independently assessed for inclusion by four authors. Any disagreements regarding study eligibility were resolved through discussion and consensus. If consensus could not be achieved, a senior author was consulted to make the final determination. The entire selection process was documented in line with the PRISMA flowchart. Inclusion and Exclusion criteria is summarized in Table 1.

### 2.4. Data Extraction

Two authors independently extracted data using a standardized extraction form. The collected data included first author, year of publication, sample size, study design, type of skin condition addressed, source of exosomes used, improvements in scar thickness, pigmentation, wrinkles, any reported adverse effects, and overall study outcomes. Any inconsistencies between the reviewers were resolved through detailed discussion and mutual agreement. When data were unclear or incomplete, corresponding authors were contacted for clarification. Findings were synthesized thematically and organized into summary tables for comparison across studies based on intervention type, outcome domain, and skin condition.

### 2.5. Risk of Bias Assessment

The risk of bias for included studies was independently assessed by two reviewers using validated tools appropriate to the study design. For randomized controlled trials (RCTs), the Revised Cochrane Risk of Bias Tool (RoB 2) was utilized [11]. This tool evaluates five fixed domains related to trial design, conduct, and reporting, using a structured set of questions. Based on responses, an algorithm generates an overall risk of bias judgment categorized as “low” (low risk in all domains), some concerns (at least one domain raises concern), or “high” (at least one domain has a high risk or multiple domains raise concerns). For non-randomized studies, the Methodological Index for Non-Randomized Studies (MINORS) tool was applied [12]. This instrument assesses methodological quality items, each scored from 0 (not reported) to 2 (reported and adequate). All studies were independently scored by two authors. Discrepancies in assessment were resolved through consensus-based discussion.

### 2.6. Data Analysis

Due to the considerable differences among the included studies, a meta-analysis could not be conducted. The presence of both clinical and methodological heterogeneity limited direct comparisons of outcomes across studies. Consequently, a qualitative synthesis was performed to systematically compile and the available evidence.

## 3. Results

**Figure 1:**
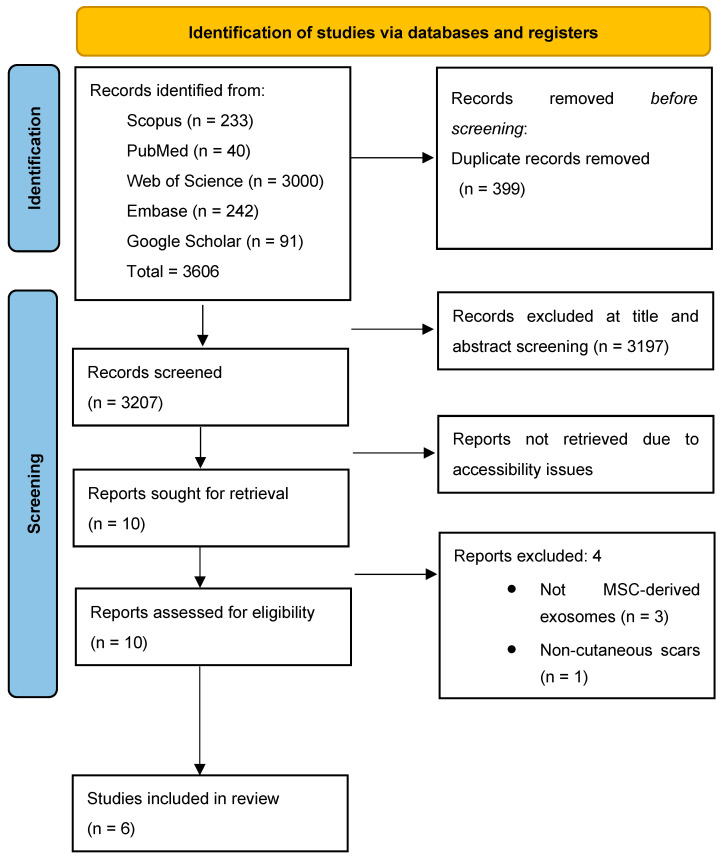
PRISMA flowchart of included studies.

The database search yielded a total of 3606 articles, of which duplicates were removed, leaving 3207 for screening. From these, 10 articles were selected for inclusion based on title and abstract screening. Ultimately, 6 articles were included in this systematic review (Figure 1 and Figure S1).

### 3.1. Study Characteristics

The systematic review included six articles investigating the efficacy and safety of MSC-exosomes were included in our study.( The included clinical studies varied not only in population characteristics and design but also in the composition and manufacturing of their exosome products. The biologic features of the exosomes, including their cellular source, particle yield, size distribution, identity markers, purity, and dosage standardization, are summarized below to ensure interpretability and cross-study comparison. The characterization criteria used in the included studies were evaluated in reference to the ISEV MISEV2018 guidelines, which outline the minimal information required for EV isolation, quantification, and molecular characterization. Most studies in this review adhered partially to these recommendations, providing NTA-based particle size measurements, morphological confirmation by TEM/cryo-TEM, and tetraspanin expression profiles. However, several studies lacked negative-marker analysis or did not fully detail isolation procedures, limiting full compliance with MISEV2018 standards [13]. To further illustrate, Table 2 summarizes which key MISEV criteria were reported in each study. Included studies were randomized controlled trials, pilot studies, case series, and case reports. A total of 99 participants with patients aged 19 years to as old as 72 years. Both males and females were represented. The summary characteristics of the included studies are reported in Table 3. The baseline features of the included studies are outlined in Table 4.

In the study by Chernoff et al. (2022) [14], the investigators employed the product XoGlo (manufactured by Kimera Labs, Miramar, FL, USA), which the company states is derived from a human placental mesenchymal stem cell (MSC) conditioned medium. According to Chernoff et al., each mL contains approximately 1 × 10^9^ exosomes. On the manufacturer’s website, the vesicles are described as having a peak size around 120–130 nm. The company reports that they use both Nanoparticle Tracking Analysis (NTA) and super-resolution dSTORM microscopy (direct Stochastic Optical Reconstruction Microscopy) for characterization of the exosome preparations. With respect to the isolation/purification method, it is not explicitly described in the published paper; however, the manufacturer reports that the conditioned medium undergoes a proprietary serial purification process and mentions the use of a Beckman ultracentrifuge. As these parameters are manufacturer-reported and not independently verified, they should be interpreted cautiously [19].

In Kwon et al. (2020) [7], adipose-derived mesenchymal stem cell exosomes were isolated from human adipose-derived stem cell–conditioned medium using the ExoSCRT purification platform. Briefly, ASCs were cultured in serum-free DMEM, and the collected conditioned medium was first passed through a 0.2 μm filter to remove cellular debris. Exosomes were then concentrated and purified using tangential-flow filtration with a 500 kDa molecular weight cut-off membrane. The resulting exosome preparation was characterized by nanoparticle tracking analysis (NTA), which confirmed a mode particle size of approximately 117 nm (within the expected 30–200 nm exosomal size range) and a concentration of 3.26 × 10^11^ particles/mL. Cryo-TEM further demonstrated the typical spherical morphology with a bilayer membrane, and bead-based flow cytometry verified the expression of standard exosomal surface markers CD9, CD63, and CD81, with low levels of calnexin and cytochrome C confirming sample purity. For clinical application, the purified exosomes were formulated into two gel preparations. On the day of fractional CO_2_ laser treatment, exosomes were applied at a concentration of 9.78 × 10^10^ particles/mL, while a lower concentration of 1.63 × 10^10^ particles/mL was used for continued application over the following days. These concentration differences were designed to support enhanced wound healing immediately after laser-induced microablative injury and to sustain regenerative effects during the subsequent recovery phase [20].

The studies in this review used diverse isolation and characterization approaches, including tangential-flow filtration (TFF), ultrafiltration, and various proprietary good manufacturing practice (GMP)–based methods, along with analytical platforms such as nanoparticle-tracking analysis (NTA), transmission electron microscopy (TEM), cryo-TEM, and bead-based flow cytometry. Several reports have compared the performance of these isolation techniques. For instance, Busatto et al. Technology demonstrated that TFF achieved higher extracellular vesicle (EV) recovery and greater consistency compared with ultracentrifugation [21], while Visan et al. (2022) similarly found that TFF yielded superior reproducibility and scalability for small-EV isolation [22]. Auger et al. (2022) also described TFF as advantageous for large-volume processing because of its continuous filtration design [23]. Chen et al. (2022) reviewed multiple exosome isolation strategies and noted that method-specific differences in selectivity and purity can influence the presence of co-isolated macromolecules, though they did not quantify the effect on potency or composition [24]. Differences in measurement techniques were also emphasized in several studies. Comfort et al. (2021) reported that NTA in light-scattering mode detects all nanoparticles in suspension, including non-vesicular particles, and therefore should be complemented by orthogonal analyses such as TEM or cryo-TEM [25].

Collectively, the literature indicates that isolation and measurement methodology strongly affect the apparent yield and properties of exosome preparations. Accordingly, variations in purification platforms and analytical tools should be considered when comparing results across studies [21,23,26,27,28]. For consistency, all exosome concentrations in this review were normalized and expressed as particle concentration (particles/mL), enabling direct comparison of dosage strength across studies with differing administration volumes.

### 3.2. Risk of Bias Assessment

**Figure 2:**
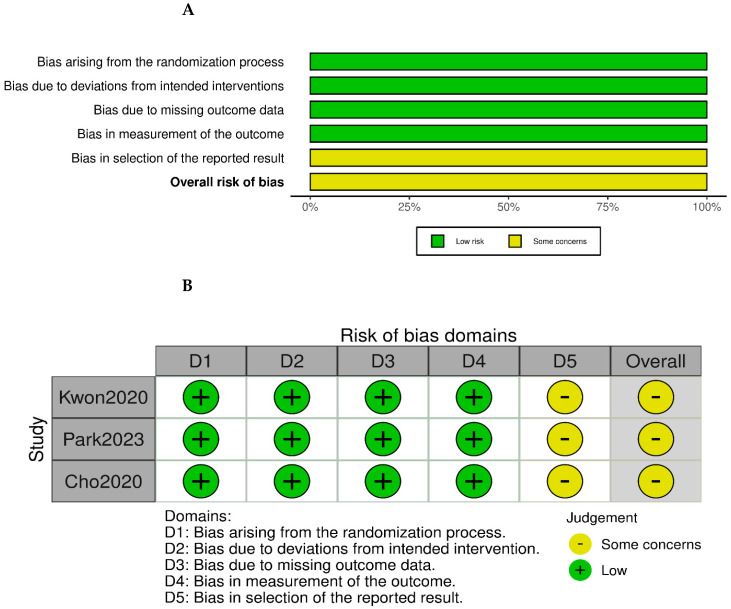
Risk of bias assessment of included studies. (**A**) Risk of bias for randomized controlled trials assessed using the ROB-II tool. (**B**) Risk of bias summary across ROB-II domains for the included randomized studies. Included studies: Kwon et al. (2020) [7], Park et al. (2023) [16] and Cho et al. (2020) [18].

The ROB II tool has been used to assess the quality of three randomized clinical trials. All studies have shown some concerns (Figure 2). However, the Minors’ checklist has been used to evaluate risk of bias of two non-randomized studies and the CARE checklist [Table S2] has been used to evaluate the risk of bias of the included case report, which is provided as Supplementary Material. Risk of bias among these studies was generally low, all studies clearly defined their purpose, recruited consecutively patients, and employed prospective data collection. However, all studies did not report on prospective sample size calculation or had an unbiased assessment of the study endpoint, which may introduce some bias. Overall, the six included studies had a low to moderate risk of bias Table 5.

### 3.3. MSC-Exosomes Characteristics

The origin of exosomes was different, including placental mesenchymal stem cells, adipose tissue stem cells, and Wharton’s jelly. Additionally, multiple isolation methods were used, such as tangential-flow filtration, GMP processing, and ultrafiltration. Moreover, multiple approaches, including direct wound injections, topical gels, slow topical application, micro needling, and subcision, were applied for exosome delivery. The exosome doses were also varied from millions to billions across the included studies. Furthermore, the included studies reported multiple pretreatment techniques, including fractional laser, fractional CO_2_ laser, chemical peels, and micro needling. The exosome features of the included studies are outlined in Table 6.

Table 7 provides a summary of clinical outcomes related to MSC-derived exosome therapies for various dermatological issues, such as keloid scars, acne scars, skin aging, hyperpigmentation, and skin injuries from trauma. Thematic trends throughout the studies examined demonstrate steady enhancements in clinical results, including scar minimization [7,14,17], pigmentation and melanin concentrations [16,18], as well as overall skin texture, moisture, and elasticity [15,16]. In studies featuring control comparisons, the side receiving the exosome treatment frequently showed statistically better results.

## 4. Efficacy

### 4.1. Efficacy in Scar Thickness

The ECCA scale (Échelle d’Évaluation Clinique des Cicatrices d’Acné) was used in Kwon et al. 2020 [7] in order to evaluate scar thickness. A significant reduction in scar thickness was noted on both sides after treatment (32.5%) in comparison with the control (19.9%) at final follow-up (*p* < 0.01). Specifically, an improvement in M-shaped scars was demonstrated after treatment with exosomes in subtype analysis. The effectiveness of exosomes in reducing scar thickness was assessed using Investigator Global Assessment (IGA) scores. A higher number of facial scars with ≥2 grades of improvement were reported on both sides with a statistically significant result (*p* = 0.02). Similarly, atrophic scar volume, pore volume, and skin surface roughness were reduced on the exosomes side compared with the control side by the 3D imaging modality. The case report by Pastrana-López 2024 [17] presented a patient with a remarkable evolution from simple acne to nodular cystic acne and scarring acne. While multiple treatment modules were applied, including dermatological procedures, changes in skincare regimen, and diet, a decrease from 180 to 90 points in the ECCA score was noted after exosomes and PRP therapy. Subsequently, these therapies improved skin tissue and reduced residual marks. The Global Aesthetic Improvement (GAIS) was used in Park et al. 2023 [16] to evaluate scar thickness. A gradual increase in the GAIS score was reported on both facial sides. Nevertheless, the exosome side revealed improvement in facial skin aging compared to the normal saline control side (*p*  =  0.005). Although there was a non-statistically significant difference between the two treatments at Week 3 (*p*  =  0.202), a statistically significant difference was demonstrated at Week 6 (*p*  =  0.023).

### 4.2. Efficacy in Hyperpigmentation

Low levels of melanin were observed in patients after twice-daily application of an ASC-exosome within 8 weeks of follow-up in a double-blind, controlled trial by Cho et al. 2020 [18]. The investigators first noted the effect of the intervention at 4 weeks. Notably, the intervention resulted in a marked skin-lightening effect on women less than 50 years old in this study. Similarly, the authors of Park et al. 2023 [16] reported a significant reduction in the melanin index on the exosomes side (9.9%) in comparison with the control side (1.0%) (*p* = 0.044).

### 4.3. Efficacy in Wrinkles, Elasticity, and Hydration

Regarding wrinkles, a great reduction in Ra, Rt, and Rz parameters was observed in Park et al. 2023 [16] (mean decrease of 12.4%, 14.4%, and 13.4%, respectively) on the intervention side in comparison with controls (*p* < 0.05). In the same experiment, the skin on the exosome side (+11.3%) was more elastic than the control side (−3.3%) (*p* = 0.002). Additionally, more hydrated skin appeared on the intervention side compared to controls (6.5% vs. 4.5%; *p* 0.037).

## 5. Pain

In the case series by Peredo et al. 2024 [15], topical human-derived exosomes were applied to 3 patients. Treatment by exosomes leads to a reduction in atrophic scar severity and skin surface roughness. Subsequently, the treatment led to a rapid reduction in pain, swelling, and redness.

## 6. Safety

### 6.1. Adverse Effects

In Kwon et al. 2020 [7], the investigators reported some adverse effects, including pain, erythema, edema, and dryness in both groups. However, these symptoms mostly subsided within five days. The erythema was less severe on the exosome side (*p* = 0.03) with shorter downtime (mean 4.1 vs. 4.3 days; *p* = 0.03).

While mild hyperpigmentation was seen in some patients, no permanent scarring was reported. In contrast, no serious complications were reported in Park et al. 2023 [16], although transient erythema, edema, and petechiae occurred. Similarly, no adverse effects were reported in Cho et al. 2020 [18].

### 6.2. Recurrence

In Chernoff et al. 2022 [14], 18 out of 21 patients with keloid scars had no recurrence after 2 years of follow-up. While three patients developed a recurrence of keloid scars within 2 months, all three recurrent keloids remained recurrence-free after six months of removal.

The safety outcomes of MSC-derived exosome therapy are detailed in Table 8. Reported adverse events were consistently mild, localized, and transient, most commonly presenting as temporary erythema, edema, procedural discomfort, dryness, or self-resolving petechiae. No infections, scarring, systemic reactions, or long-term treatment-related complications were documented across studies. Follow-up duration varied, with the longest observation extending to 2 years [14], while the remaining studies evaluated short-term safety over 6–12 weeks. While findings indicate a favorable short-term safety profile, the limited long-term follow-up in most trials underscores the need for extended monitoring in future controlled studies.

## 7. Discussion

This systematic review presents a focused evaluation of the clinical efficacy of mesenchymal stem cell-derived exosomes (MSC-exosomes) in treating common dermatological conditions such as scars, hyperpigmentation, and skin aging. Across six human studies involving a total of 99 participants (63 females and 36 males) from diverse geographic regions (United States, Republic of Korea, and Mexico), MSC-exosome therapy demonstrated promising results in both aesthetic improvement and regenerative healing, even though the research on this is still in its infancy. Although mesenchymal stem cells (MSCs) themselves have been well-characterized as a potent therapy, they can even be improved upon by isolation of their cell-free derivative secretome and, in particular, exosomes.

A significant finding of this review was the overall enhancements in skin texture, hydration, elasticity, and pigmentation appearance after MSC-exosome treatment [7,14,15,16,17,18]. The clinical benefits mirror what we know about the functions of exosomes, including their part in intercellular communication, inflammation modulation, tissue repair, and cellular rejuvenation [6,7]. In particular, RCTs show the thickness of atrophic acne scars reduced by 32.5% in the group treated with exosomes, compared to 19.9% in the control group (*p* < 0.01) [7]. Aging skin measurements like Ra, Rt, and Rz, which show skin texture, improved by 12.4%, 14.4%, and 13.4% in the exosome group, compared to 6.6%, 6.8%, and 7.1% in the control group (*p* < 0.05). Skin elasticity increased by +11.3% versus −3.3% in the control group (*p* = 0.002), indicating enhanced dermal resilience. Hydration levels also improved more in the exosome group, with a 6.5% increase versus 4.5% in the control group (*p* = 0.037). Exosomes also led to a more significant reduction in the melanin index, decreasing by 9.9% compared to just 1.0% in the control group (*p* = 0.044) [16], corresponding to visible aesthetic improvement within 6 weeks of therapy. Scar-type analysis revealed complete resolution of scars and partial improvement in boxcar/rolling scars, alongside significant clearance of post-inflammatory hyperpigmentation. Subjectively, patients reported high satisfaction, including descriptions of “significant disappearance of acne.” [17]. Across all studies, the side effects were mild and temporary, such as redness, swelling, or darkening of the skin, and no serious long-term safety issues were reported. The clinical findings across included studies indicate that the method of exosome delivery substantially influenced treatment outcomes. In Chernoff et al. (2022), direct intralesional injection of placental MSC-derived exosomes into the wound base resulted in deep tissue penetration and produced a very low keloid recurrence rate over two years, suggesting enhanced stability and regenerative signaling at deeper dermal levels [14]. In contrast, Kwon et al. (2020) used topical adipose-derived exosomes applied immediately after fractional CO_2_ laser, where the laser-created microchannels significantly improved transdermal absorption, resulting in greater reductions in atrophic scar volume, pore size, and roughness compared to control [7]. Similarly, Park et al. (2023) demonstrated that microneedling-assisted topical delivery produced superior improvements in wrinkles, elasticity, hydration, and pigmentation, further supporting that pretreatment devices enhance exosome penetration [16]. Peredo et al. (2024) showed that slow, controlled topical infusion after aesthetic procedures accelerated healing and reduced pain, swelling, and erythema [15]. Meanwhile, Pastrana-López (2024) reported that deep dermal cannula infiltration, combined with subcision, yielded marked improvements in acne scarring, including complete resolution of scars [17].

This is more apparent in the backdrop of previous preclinical studies that support these observations. For example, Yuan et al. (2023), in the animal study of adipose tissue-derived stem cell (ADSC) exosome management, have demonstrated that the quality of wound closure, vascularization, and overall healing is substantially improved in animals that receive ADSC-derived exosomes compared to those that receive control saline [29]. Similarly, a systematic review and meta-analysis conducted in 2025 by Zhu et al. [30] provided strong evidence from preclinical studies showing that mesenchymal stem cell-derived extracellular vesicles (MSC-EVs) are effective in treating wounds and helping skin repair. They combined data from 83 studies and found that MSC-EVs help wounds close faster, increase collagen production, and improve blood vessel formation in both diabetic and non-diabetic animal models. MSC-EVs help diabetic individuals heal their wounds by both stimulating and preventing vital biological functions. They encourage angiogenesis (the creation of new blood vessels), differentiation, and cell proliferation, all of which are critical for tissue healing. At the same time, they prevent detrimental processes like inflammation, fibrosis (the creation of scar tissue), and apoptosis (the death of cells) [30].

In contrast to the consistently positive outcomes observed in the present review, several other publications have reported more variable or limited effects of MSC-exosome therapy. For example, the included studies in our review [7,14,15,16,17,18] show improvements in skin texture, pigmentation, and elasticity, while Ong et al. [31] confirmed that MSC-exosomes contribute to immune modulation and tissue repair in corneal wounds but noted that these effects may not be directly translatable to cutaneous tissues given the fundamental differences in vascularity and epithelial turnover.

This systematic review demonstrates several methodological strengths that enhance its validity and contribution to the field. First, screening and data extraction were performed in a blinded manner by independent reviewers, minimizing the risk of bias. Second, the inclusion criteria were deliberately structured to capture a wide range of study designs, including randomized controlled trials, split-face trials, and case series. Finally, the inclusion of studies from diverse geographical contexts, including the United States, the Republic of Korea, and Mexico, enhances the global relevance and applicability of the findings.

Although the review uses a strong research approach, there is a limitation due to the differences in the quality and design of the studies included. These differences made it challenging to compare the studies directly and perform a meta-analysis. In addition, the scarcity of human clinical trials required the inclusion of case reports and case series, which provide lower levels of evidence and limit the overall strength and applicability of the conclusions. Although MSC-exosome therapy shows considerable potential for treating skin conditions, significant unanswered questions indicate that it requires more comprehensive clinical research. Future research should prioritize large, well-designed randomized controlled trials with follow-up periods extending beyond one year to determine the durability of benefits and assess long-term safety. In addition, comparative studies are needed to evaluate whether factors such as exosome source, dosage, and delivery method influence healing rates and scar quality, enabling a more standardized and optimized therapeutic approach. To elucidate, differences in isolation, purification, and characterization methods constitute a substantial source of heterogeneity and should be explicitly acknowledged when interpreting comparative efficacy and safety across studies. While the studies included all showed overall improvements in skin texture, pigmentation, and elasticity, the certainty of evidence is overall moderate. The RoB 2 assessment found “some concerns” in most randomized trials, mainly as a consequence of unclear randomization and a lack of blinding, whereas the MINORS evaluation suggested moderate quality among non-randomized studies. To clarify the evidence hierarchy relevant to our findings, randomized controlled trials represent the highest level of evidence, non-randomized studies provide moderate evidence, and single case reports offer the lowest level; therefore, the conclusions of this review should be interpreted with appropriate caution.

## 8. Conclusions

In conclusion, mesenchymal stem cell (MSC)–derived exosomes show promising benefits for improving scars, hyperpigmentation, and skin aging, with reported reductions in scar thickness, pigmentation, pain, and wrinkle severity, as well as improvements in skin elasticity and hydration, and generally minimal adverse effects. However, these findings should be interpreted with caution. The overall evidence remains preliminary, limited by the small number of human clinical studies, heterogeneity in study designs, variations in exosome source and dosing, and relatively short follow-up durations. Although interest in the dermatologic application of exosomes is rapidly growing, much of the existing support still comes from laboratory and animal research rather than robust clinical trials. Larger, well-designed randomized controlled trials with standardized protocols and extended follow-up are needed to clarify the long-term safety, durability, and true clinical efficacy of MSC-derived exosomes. Future investigations should also aim to optimize exosome sources, concentrations, delivery methods, and treatment protocols to ensure reproducible and clinically meaningful outcomes. Overall, while current human data suggest meaningful potential, MSC-derived exosomes should be regarded as an emerging therapy that requires further validation before widespread clinical adoption.

## Figures and Tables

**Table 1:** Inclusion and Exclusion Criteria.

Inclusion Criteria	Exclusion Criteria
Population: Human participants of any age/gender with cutaneous skin conditions such as scars, aging and hyperpigmentation	Studies will be excluded if they involve non-human subjects, including in vitro (lab-based) or animal models.
Studies will be considered if they assess the use of mesenchymal stem cell (MSC)-derived exosome therapies, delivered either topically or via injection, for the purpose of scar treatment.	Articles will also be excluded if they involve non-cutaneous scars, such as scarring of internal organs (e.g., cardiac, liver, or lung fibrosis).
Studies reporting at least one clinical outcome related to scar improvement, Pigmentation improvement, Anti-aging effects (e.g., texture, thickness, pigmentation, wrinkle depth, melanin index and patient satisfaction).	Outcomes: Studies not reporting clinical outcomes related to scar healing, Pigmentation improvement, Anti-aging effects
Study Design: Clinical trials (randomized or non-randomized), cohort studies, case series, or case reports, Pilot study.	Study Design: Reviews, editorials, opinion papers, protocols, or conference abstracts without full text.
Language: Articles published in English.	Language: Articles not published in English.
Publication Date: Articles published from 2010 to 2025.	Any studies that utilize non-MSC-derived exosomes (e.g., platelet-derived or tumor-derived exosomes)

**Table 2:** Reporting of Key MISEV Criteria in Included Articles.

Study	Particle Size (Reported)	Quantification (Particles/mL)	Positive Markers	Negative Markers	Purity/Isolation Description (Reported)
**Chernoff et al., 2022** [14]	Peak ~120–130 nm (according to manufacturer)	5 billion exosomes in 5 mL (initial); additional 3 billion exosomes (1.0 mL) for first 3 weeks	N/A	N/A	Isolation not explicitly described in paper; manufacturer reports proprietary serial purification and Beckman ultracentrifuge
**Kwon et al., 2020** [7]	Mode ~117 nm (measured via NTA)	3.26 × 10^11^ particles/mL (NTA); clinical doses: 9.78 × 10^10^ and 1.63 × 10^10^ particles/mL	CD9, CD63, CD81 (bead-based flow cytometry)	Low calnexin and cytochrome C	ExoSCRT™; 0.2 μm filtration then tangential-flow filtration (500 kDa MWCO)
**Peredo et al., 2024** [15]	N/A	2–5 × 10^9^ and 4.8 × 10^9^ particles/mL	N/A	N/A	Exovex™ processed under GMP with QC testing (NTA, NGS, MudPIT)
**Park et al., 2023** [16]	N/A	N/A	N/A	N/A	ExoSCRT™ platform mentioned.
**Pastrana-López, 2024** [17]	N/A	3 × 10^10^ particles/unspecified	N/A	N/A	Isolation method: N/A/Not stated in paper; delivery: infiltration with 25 G cannula + subcision
**Cho et al., 2020** [18]	30–200 nm	1.47 × 10^12^ particles/mL isolated -clinical dose not specified	CD9, CD63, CD81	calnexin	tangential flow filtration (TFF) using a 100 kDa molecular weight cutoff membrane cartridge with phosphate-buffered saline

**Table 3:** Study characteristics of included studies.

Study ID (Author, Year)	Country	Journal	Study Design	Participants (N)		
				Total Number of Participants	Female	Male
**Chernoff, 2022** [14]	United States	Journal of Surgery	pilot study	21	11	10
**Kwon, 2020** [7]	Republic of Korea	Acta Dermato-Venereologica	Randomized Controlled Trials	25	7	18
**Peredo, 2024** [15]	United States	Journal of Drugs in Dermatology	Case Series	3	3	0
**Park, 2023** [16]	Republic of Korea	Journal of Cosmetic Dermatology	prospective, randomized, split-face, comparative study	28	20	8
**Pastrana-López, 2024** [17]	México	Journal of Stem Cell Research	Case Report	1	1	0
**Cho, 2020** [18]	Republic of Korea	Cosmetics	Prospective, split-face, double-blind, randomized placebo-controlled study	21	21	0

**Table 4:** Baseline characteristics of included studies.

Study ID (Author, Year)	Range of Age (Years)	Skin Condition (Scar, Aging, Hyperpigmentation)
**Chernoff, 2022** [14]	23–57	Keloid scar
**Kwon, 2020** [7]	19–54	Atrophic acne scars
**Peredo, 2024** [15]	31–72	Acne, melasma, aging, dog bite trauma
**Park, 2023** [16]	43–66	Facial aging (features: wrinkles, elasticity loss, irregular pigmentation)
**Pastrana-López, 2024** [17]	33 year-old female patient	Severe acne scars (boxcar, rolling scars) and hyperpigmentation.
**Cho, 2020** [18]	39–55	Hyperpigmentation (focused on melanin reduction and skin brightening).

**Table 5:** Risk of Bias assessment using Minors’ checklist.

Study ID (Author, Year)	1. A Clearly Stated Aim	2. Inclusion of Consecutive Patients	3. Prospective Collection of Data	4. Endpoints Appropriate to the Aim of the Study	5. Unbiased Assessment of the Study Endpoint	6. Follow-Up Period Appropriate to the Aim of the Study	7. Loss to Follow Up Less than 5%	8. Prospective Calculation of the Study Size	Total
**Chernoff 2022** [14]	**2**	**2**	**2**	**2**	**0**	**2**	**2**	**0**	**12**
**Peredo 2024** [15]	**2**	**2**	**2**	**2**	**0**	**2**	**2**	**0**	**12**

**Table 6:** Exosome Features of the included studies.

**Study ID**	Concentration of Exosome	Isolation Method	Source of Exosome	Exosome Delivery Method
**Chernoff, 2022** [14]	1 × 10^9^ particles/mL	GMP-certified tangential-flow filtration (TFF) process	Human placental, fetal mesenchymal stem cells (XoGlo)	Direct injection into wound base
**Kwon, 2020** [7]	9.78 × 10^10^ particles/mL	ExoSCRT™, Exosomes were isolated from ASC-conditioned medium using filtration and tangential-flow purification, then quantified by nanoparticle tracking analysis (NTA).	Adipose tissue-derived mesenchymal stem cells	Topical gel applied immediately post-laser and twice daily for 2 days post-treatment
**Peredo, 2024** [15]	2–5 × 10^9^ and 4.8 × 10^9^ particles/mL	Exovex™, Processed under GMP with QC testing (NTA, NGS, MudPIT)	Human placental mesenchymal stem cells	Topical application (Cases 1, 2); slow topical delivery using 32 G needle (Case 3)
**Park, 2023** [16]	N/A	ExoSCRT™	Adipose tissue-derived mesenchymal stem cells	Step 1: Microneedling (1.0 mm depth) performed first to create microchannels. Step 2: 2 mL of HACS (exosome solution) applied topically immediately after microneedling to the treatment side.
**Pastrana-López, 2024** [17]	1 × 10^10^ particles/mL	N/A	Derived from umbilical cord mesenchymal stem cells from Wharton’s jelly	Infiltration with a 25 G cannula into the deep dermal plane. Combined with subcision for mechanical release of fibrous bands.
**Cho, 2020** [18]	1.47 × 10^12^ particles/mL isolated however clinical dose used not specified	Exosomes were isolated from cultured ASCs using tangential flow filtration, stored at −80 °C, and characterized by nanoparticle tracking analysis	Adipose tissue-derived mesenchymal stem cells	Topical application (cosmetic formulation: gel with glycerin, xanthan gum, Carbopol 344)

Abbreviations: QC = Quality Control, NTA = Nanoparticle Tracking Analysis, NGS = Next-Generation Sequencing, MudPIT = Multidimensional Protein Identification Technology.

**Table 7:** Clinical outcomes, Adverse effects, and Key findings.

Study ID	Outcomes		Adverse Effects	Key Findings
	Intervention Side Outcome	Control Side Outcome		
**Chernoff, 2022** [14]	18 out of 21 patients had no recurrence of keloid scars over a 2-year follow-up period.	N/A	No adverse effects were reported.	1. Post-inflammatory hyperpigmentation occurred in Fitzpatrick skin types III–VI. 2. Clinical results suggest exosome therapy may reduce keloid recurrence.
**Kwon, 2020** [7]	32.5% Reduction	19.9% Reduction	No scarring or permanent adverse effects were reported.	1. Better Global Assessment: Based on Investigator Global Assessment (IGA), 16/25 exosome treated sides achieved ≥ grade 2 improvement, compared to 12/25 control sides (*p* = 0.02), indicating greater visible improvement. 2. Objective Skin Improvements (3D Imaging): Only the exosomes treated sides showed significant reductions in atrophic scar volume, pore size, and skin roughness.
**Peredo, 2024** [15]	Rapid healing, reduced swelling, erythema, pain, scarring across all cases	N/A	No adverse effects were reported.	Case 1 (31-year-old, melasma): Non-ablative fractional laser + 3 mL exosomes reported reduced erythema and pain (from 8/10 to 4/10). Case 2 (72-year-old): Prior CO2 laser + PRP = 1-week erythema/swelling; later repeat with exosomes (2.5 mL) = reduced erythema/swelling by Day 4. Case 3 (49-year-old): Dog bite to lower lip, treated with 2.5 mL exosomes slowly over 10 min; complete wound closure by Day 10 (vs. 6-month estimated healing time), minimal scarring, preserved function.
**Park, 2023** [16]	Wrinkles: improved by 12.4%, 14.4%, and 13.4% (*p* < 0.05), Elasticity: Increased by 11.3% (*p* = 0.002), Hydration: Rose by 6.5% (*p* = 0.037), Melanin index dropped by 9.9% (*p* = 0.044)	Wrinkles: decreased by 6.6%, 6.8%, and 7.1%, Elasticity: Declined by 3.3%, Hydration Increased by 4.5%, Melanin index reduced by 1.0%	Mild erythema, temporary swelling, minor bleeding resolved quickly, no serious adverse effects.	1. The Global Aesthetic Improvement Scale (GAIS) score was significantly higher on the exosome treated side than on the control side at the final follow-up visit (*p* = 0.005). 2. Superior Anti-Aging Effects: xosome + microneedling significantly improved wrinkles, elasticity, hydration, and pigmentation compared to saline control. 3. Histological Confirmation:-Increased collagen, elastic fibers, and mucin in treated skin, supporting clinical results.
**Pastrana-López, 2024** [17]	Significant improvement in skin appearance; ECCA score decreased from 180 to 90 points; regeneration of scar tissue; improved skin texture and color.	no control group	No adverse effects were reported.	1. Scar Type-Specific Results: -Ice-pick scars: Complete resolution -Boxcar/Rolling scars: Partial improvement 2. Hyperpigmentation Reduction -Post-inflammatory hyperpigmentation: Significant clearance 3. Subjective Outcomes -Implied Satisfaction: Patient reported “significant disappearance of acne”
**Cho, 2020** [18]	Statistically significant reduction in melanin levels compared to placebo control, starting from 2 weeks with peak efficacy at 4 weeks	No significant reduction in melanin levels compared to the intervention side.	No adverse effects were observed in any volunteer during or after the study.	1. The effect was more pronounced in volunteers aged < 50 years. 2. Melanin reduction was transient, diminishing by 8 weeks, suggesting limited long-term efficacy.

Abbreviations: ECCA: (Échelle d’Évaluation Clinique des Cicatrices d’Acné).

**Table 8:** Safety Profile and Follow-Up Duration of Included Studies.

Study (First Author, Year)	Follow-Up Duration	Reported Adverse Events	Severity/Description
**Chernoff, 2022** [14]	2 years	No adverse effects reported	No treatment-related complications
**Kwon, 2020** [7]	12 weeks (6-week post-final assessment)	Pain, erythema, edema, dryness	Common, transient, resolved without sequelae
**Peredo, 2024** [15]	Up to 10 days (case-based, short-term)	No adverse effects reported	No safety concerns noted
**Park, 2023** [16]	6 weeks after last treatment	Mild erythema, swelling, petechiae	Self-limiting, resolved spontaneously
**Pastrana-López, 2024** [17]	45–60 days between sessions (clinical follow-up)	No adverse effects reported	No complications observed
**Cho, 2020** [18]	8 weeks	No adverse effects observed in any participant	No local or systemic adverse reactions

## Data Availability

All data analyzed in this review are available in the published articles listed in the references.

## Supplementary Materials

The following supporting information can be downloaded at: https://www.mdpi.com/article/10.3390/reports8040268/s1, Figure S1: Prisma Flowchart; Table S1: Prisma 2020 checklist, Table S2: CARE checklist for case reports.

## Abbreviations

MSCs	Mesenchymal Stem Cells
MSC-Exos	Mesenchymal Stem Cell–Derived Exosomes
MSC-EVs	Mesenchymal Stem Cell–Derived Extracellular Vesicles
ADSC	Adipose tissue-derived Stem Cell
RCT	Randomized Controlled Trial
PRISMA	Preferred Reporting Items for Systematic Reviews and Meta-Analyses
GMP	Good Manufacturing Practice
NGS	Next-Generation Sequencing
MudPIT	Multidimensional Protein Identification Technology
ECCA	Échelle d’Évaluation Clinique des Cicatrices d’Acné
GAIS	Global Aesthetic Improvement Scale
IGA	Investigator Global Assessment
RoB 2	Revised Cochrane Risk of Bias Tool
MINORS	Methodological Index for Non-Randomized Studies
NTA	Nanoparticle Tracking Analysis
